# Supportive care needs and associated factors among caregivers of patients with colorectal cancer: a cross-sectional study

**DOI:** 10.1007/s00520-024-08390-w

**Published:** 2024-02-27

**Authors:** Menghan Zhang, Xin Wang, Mengwei Shao, Ruofei Du, Huiyue Zhou, Jizhe Zhu, Haoning Zhang, Bin Ma, Changying Chen, Tao Wang

**Affiliations:** 1 The College of Nursing and Health of Zhengzhou University, Zhengzhou, 450001 China; 2 Academy of Medical Sciences of Zhengzhou University, Zhengzhou, 450001 China; 3 Sun Yat-Sen Memorial Hospital, Sun Yat-Sen University, Guangzhou, 510000 China; 4 School of Medical, Molecular and Forensic Sciences, Murdoch University, Perth, 6149 Australia; 5 Department of Quality Control, The First Affiliated Hospital of Zhengzhou University, Zhengzhou, 450052 China; 6 Telethon Kids Institute, Perth, WA 6872 Australia; 7 Medical School, University of Western Australia, Perth, WA 6872 Australia

**Keywords:** Colorectal cancer, Caregiver, Supportive care needs, Caregiver preparedness, Benefit finding, Financial toxicity

## Abstract

To assess the level of supportive care needs of caregivers of colorectal cancer patients and explore the related key influencing factors. Totaling 283 caregivers of patients with colorectal cancer were investigated in this study. Firstly, caregivers were invited to complete a set of questionnaires, including the general information questionnaire, the Supportive Care Needs Survey-Partners and the Caregivers of cancer patients, the Caregiver Preparedness Scale, the Benefit Finding Scale, and the Comprehensive Score for Financial Toxicity. Univariate and multivariate linear regression were performed to investigate the associated factors of supportive care needs. The caregivers of patients with colorectal cancer have a moderate level of needs, scored at 2.71 ± 0.42. Caregiver preparedness, benefit finding, and financial toxicity were significantly negatively associated with the supportive care needs of caregivers (*r* =  − 0.555, *P* < 0.001; *r* =  − 0.534, *P* < 0.001; and *r* =  − 0.615, *P* < 0.001, respectively). Our multivariate regression analysis identified some factors that directly affected the supportive care needs of caregivers, including the duration of illness, tumor stage, the age and educational level of caregivers, caregiver preparedness, benefit finding, and financial toxicity (*R*^2^ = 0.574, *F* = 23.337, *P* < 0.001). Supportive care needs are common among caregivers of colorectal cancer patients. Higher caregiver preparedness, benefit finding, and financial toxicity tend to ease these needs. Healthcare workers should have an in-depth understanding of the needs of caregivers of colorectal cancer patients and actively provide targeted financial/informational/technical/emotional support to promote nursing skills and reduce caregivers’ burdens.

## Introduction

Colorectal cancer (CRC) is the third most common cancer worldwide and the second most common cause of cancer-related death [1]. The latest global cancer data shows more than 1.90 million cases and 935,000 deaths yearly, accounting for 10% of all cancer incidences [2]. In the last 10 years, although the CRC mortality rate has decreased, CRC incidence among young and middle-aged adults under 50 is rising by nearly 2% per year [3].

Currently, the main treatment methods for CRC are surgery, chemotherapy, targeted therapy, and the recently developed immunotherapy [4]. Although the 5-year survival rate for patients with localized colorectal cancer has dramatically increased to more than 90% in recent years, CRC patients face long-term challenges (e.g., post-surgery intestinal function changes) due to ostomy, accompanied by fatigue, insomnia, and poor stomach tolerance [5]. A recent study showed that in addition to helping patients change their colostomy bag, paying attention to nutritional status and preventing complications also cause significant caregiver burdens [6, 7]. In another case, it was found that caregivers could also experience social isolation due to heavy caregiving tasks, which change their lifestyles (reduced recreational activities and personal lives) [8]. Indeed, the prolonged treatment cycle, complex treatment process, multiple adverse reactions, and high medical costs cause distress to patients and bring physical and psychological burdens to their caregivers [8, 9]. These resulted in a high level of Supportive Care Needs (SCN), which refers to the services needed by both cancer patients and their caregivers during cancer care [10].

As reported by recent studies, the caregivers of CRC patients generally reported a high level of psychological distress (e.g., anxiety, depression, fear, and guilt) [11]. This was because CRC patients were more dependent on nursing/caring services (due to the high incidence of stoma complications) [12]. As demonstrated by a study led by Pape, caregivers experienced comparable loneliness, embarrassment, and stigma as CRC patients due to colostomy [8]. As the heavy physical and psychological burdens experienced by caregivers inevitably affect how they look after patients [12], it is necessary to comprehensively analyze the difficulties caregivers face with CRC patients and identify the key factors affecting caregivers’ SCN. This is also important for enacting effective strategies to improve the quality of CRC management.

Although two critical factors of SCN, including patients’ clinical characteristics and caregivers’ sociodemographic factors, have been identified in previous studies [13–15], the following three factors may also play crucial roles in the level of SCN. The first is caregiver preparedness, defined as the perceived readiness of caregivers for the tasks of the caregiving role, such as fulfilling the physical and emotional needs of patients, planning care procedures, and managing clinical caring-related stresses [16]. The second is financial toxicity, i.e., cancer treatment’s financial burden and the resulting stress experienced by patients and caregivers [17]. The last is benefit finding, the ability of caregivers to perceive personal, social, psychological, and spiritual benefits from adverse life events (to see the bright side). A more positive attitude could protect caregivers from negative psychological impacts, guaranteeing lower burden and better quality of life [18, 19].

Although some previous studies have examined the SCN of caregivers, most of these studies focused on caregivers of patients with other types of cancers, such as lung cancer, breast cancer, and head and neck cancer, while rare research has been performed on caregivers of colorectal cancer [20]. Consequently, our study aims to (i) investigate the SCN of caregivers of CRC patients, (ii) analyze the correlation of caregiver preparedness, benefit finding, and financial toxicity with the SCN in CRC caregivers, and (iii) identify influencing factors associated with the SCN of caregivers of CRC patients. This study will improve the understanding of the SCN and influencing factors of CRC patients’ caregivers and provide a scientific basis for developing targeted interventions to meet caregivers’ needs and improve the outcomes of CRC caring.

## Methods

### Design and sample

This was a single-institution, cross-sectional pilot study. Data were collected in the First Affiliated Hospital of Zhengzhou University, Henan Province, China, from October 2022 to May 2023. Three hundred caregivers of CRC patients were recruited. The inclusion criteria were as follows: (1) caregivers who were caring for patients of primary colorectal cancer (if there were two or more caregivers, the one undertaking the primary caregiving responsibility was included); (2) aged above 18 years; (3) cared more than 8 h per day; (4) were able to read and write Chinese; (5) had given informed consent. Caregivers with any of the following conditions were excluded: (1) receive remuneration; (2) cancer, cognitive disorder, or mental illness; (3) participate in other similar trials; (4) incomplete information.

Caregivers were recruited during cancer treatment in the hospital. Doctors and nurses at the hospital were contacted in advance, and with their assistance, the caregivers of CRC patients were evaluated. We explained the purpose of the investigation to caregivers who met the inclusion criteria. After obtaining the caregivers’ consent, we distributed questionnaires and informed consent forms to them. For caregivers who had questions about the questionnaire items, we interpreted these items in neutral, non-suggestive language. Among the 300 caregivers being initially recruited, 10 refused to participate in the study because (1) they thought the research would not help them, (2) they were inconvenient, or (3) their families disagreed.

The sample size was calculated using the formula: $N=\frac{4{U}_{\alpha }^{2}{s}^{2}}{{\delta }^{2}}$. The standard deviation of SCN was evaluated in a previous study, which was 0.61 [21]. With a 95% confidence level, a margin of error of 0.05, a limit error of 0.2, and an assumed 20% invalid completion rate. We estimated that at least 179 participants should be recruited.

### Data collection and measures

#### The general data questionnaire

The general data questionnaire was designed by reviewing previous publications and consulting experts in cancer nursing. The available information in the questionnaire includes two parts. The first is the patient’s disease status: duration of illness, tumor stage, tumor metastasis, treatment method, stoma status, and type of medical insurance. The second is general information on caregivers: gender, age, place of residence, marital status, educational level, occupation, per capita family income, health status, relationship with patients, whether they live with patients, and the number of caregivers.

### Supportive care needs survey-partners and caregivers of cancer patients

The SCNS-P&C was developed by Girgis in 2011 [22]. In this study, the Chinese version of the scale modified by Liu Jingjing was adopted, with a total of 45 items [21]. Items are grouped into four need domains: health care service and information needs (16 items), psychological and emotional needs (8 items), communication and relationship needs (10 items), and social security and work needs (11 items). Respondents were asked to assess their needs with each item over the past 1 month on a 5-point Likert scale (1 = no need; 2 = need satisfied; 3 = low need; 4 = moderate need; 5 = high need), with a higher score indicating a higher level of SCN. The Cronbach’s *α* coefficient of the Chinese version SCNS-P&C was 0.946, and the Cronbach’s *α* coefficient of each dimension was 0.795 ~ 0.898, indicating good reliability [21]. In this study, the Cronbach’s *α* coefficient of this scale was 0.906.

### Preparedness for caregiving scale (PCS)

The Preparedness for Caregiving Scale (PCS) was developed to measure caregiver preparedness (how caregivers expect the associated tasks and the stress) [23]. In this study, the Chinese version of the scale developed by Liu Yanjin was adopted, with 8 items [24]. This self-report instrument consists of 8 items that represent the domains of caregiving, such as attending to physical and emotional needs, setting up support services, coping with the stress of caregiving, responding to emergencies, and accessing resources and information. The Likert 5-level scoring method was adopted, with 0–4 points from “very inconsistent” to “very consistent”, with higher scores indicating higher preparedness. The Cronbach’s *α* coefficient of the original scale was 0.925. The Cronbach’s *α* coefficient in our study was 0.945.

### Benefit finding scale

The Benefit Finding Scale (BFS) scale was compiled by Antoni et al. [25]. The revised Chinese version of the scale included 22 items in five dimensions: acceptance, family relationship, personal growth, social relationship, and health behavior [26]. The Likert 5-level scoring method was used to score 1–5 points from “none at all” to “very much” and was applied to family caregivers of cancer patients. The Cronbach’s *α* coefficient was 0.933. In this study, the Cronbach’s *α* coefficient of this scale was 0.979.

### Comprehensive scores for financial toxicity based on the patient-reported outcome measures

This scale contains 11 items in two dimensions, positive wealth status and adverse psychosocial response, and adopts the Likert 5-level scoring method, from “none at all” to “very much”, scoring 0–4 points successively, with a total score of 0–44 points [27]. According to the score, financial toxicity was divided into two grades: high financial toxicity (≤ 22 points) and low financial toxicity (> 22 points). The lower the score represented, the more severe the financial toxicity. The scale used to assess patients’ perceived stress about their financial and work situations had a Cronbach’s *α* coefficient of 0.889. Sadigh et al. applied this scale to caregivers of cancer patients and measured the Cronbach’s *α* coefficient to be 0.910 [17]. In this study, the Cronbach’s *α* coefficient of this scale was 0.972.

### Statistical analysis

The collected questionnaires were uniformly coded and checked by two investigators before entering the data. The IBM SPSS Statistics for Windows (version 26.0) was used for statistical analysis. All significant tests were two-tailed with a significance level of *P* < 0.05. Continuous data were expressed as $\overline{x }$±s, and categorical data were expressed as percentages and frequencies. The normal distribution of SCNS-P&C scores was assessed with the Shapiro–Wilk normality test. As appropriate, we compared social-demographics using the *t* test and ANOVA. Pearson correlations were applied to identify correlations between supportive care demand, caregiver preparedness, and perceived benefit of disease.

Multiple linear regression was adjusted for confounders to examine the correlation of social-demographic, caregiver preparedness, benefit finding, and financial toxicity with the SCN in CRC caregivers. To identify the significant factors associated with the total SCNS score, variables with a *P* < 0.05 in the univariate analysis were included in multivariate linear stepwise regression models, with all classification variables being changed to dummy variables.

We ensure this is an independent study. All the data/results in this article are not shown in other publications or derived from secondary analysis.

### Ethics approval

This study complied with the Declaration of Helsinki and the “Good Clinical Practice” guidelines and was approved by the Life Science Ethics Review Committee of Zhengzhou University (permit number: ZZUIRB2023-096). All caregivers provided signed informed consent before enrollment in the study.

## Results

### Participants’ characteristics

A total of 300 caregivers of CRC patients were recruited. Among them, 290 caregivers returned the questionnaires, with a response rate of 96.67%. We excluded 7 caregivers whose patients’ electronic medical record was incomplete. Eventually, there were 283 subjects included. Caregiver’s age was 46.80 ± 12.40 years (range 18 ~ 75 years). The participants included 138 males (48.8%) and 145 females (51.2%). Other detailed socio-demographics of the participants are presented in Table 1.

**Table 1:** Socio-demographics and the associations with the SCNS-P&C score (*n* = 283)

Variables	*n* (%)	SCNS-P&C score ($\overline{x }$±s)	*F/t*	*P*
Duration of illness			5.255^a^	< 0.001
< 1 month	64 (22.9)	2.74 ± 0.45		
1 ~ 6 months	123 (44.2)	2.76 ± 0.40		
6 ~ 12 months	35 (12.9)	2.82 ± 0.38		
1 ~ 5 years	58 (19.1)	2.53 ± 0.41		
> 5 years	3 (0.9)	2.19 ± 0.55		
Tumor stage			4.326^a^	0.005
I	16 (5.0)	2.40 ± 0.37		
II	229 (81.5)	2.37 ± 0.42		
III	28 (9.6)	2.63 ± 0.43		
IV	10 (3.8)	2.940.35 ±		
Tumor metastasis			− 1.236^b^	0.218
No	212 (74.5)	2.70 ± 0.44		
Yes	71 (25.5)	2.76 ± 0.37		
Cancer state			− 0.906^b^	0.366
First	263 (92.7)	2.70 ± 0.43		
Relapse	20 (7.3)	2.79 ± 0.33		
Treatment method			1.066^a^	0.364
Surgery	41 (14.1)	2.65 ± 0.42		
Chemotherapy	27 (9.8)	2.79 ± 0.41		
Other	32 (11.6)	2.79 ± 0.44		
Combined therapy	183 (64.4)	2.70 ± 0.42		
Colostomy			0.572^a^	0.656
No	204 (71.7)	2.70 ± 0.42		
Permanent colostomy	19 (6.7)	2.68 ± 0.44		
Temporary colostomy	60 (21.6)	2.76 ± 0.44		
Medical insurance			25.203^a^	< 0.001
UEBMI	63 (20.2)	2.46 ± 0.42		
URRBMI	205 (75.2)	2.81 ± 0.39		
SMI + CMI	15 (4.7)	2.38 ± 0.36		
Caregiver gender			0.044^b^	0.965
Male	138 (48.8)	2.71 ± 0.36		
Female	145 (51.2)	2.71 ± 0.48		
Caregiver age			4.778^a^	0.009
18 ~	79 (26.9)	2.61 ± 0.41		
40 ~	154 (55.8)	2.78 ± 0.40		
60 ~	50 (17.2)	2.64 ± 0.49		
Place of residence			9.155^b^	< 0.001
Rural	197 (73.1)	2.84 ± 0.36		
Urban	86 (26.9)	2.40 ± 0.41		
Marital status			1.307^a^	0.272
Unmarried	11 (3.6)	2.51 ± 0.45		
Married	271 (96.0)	2.72 ± 0.42		
Widowed or divorced	1 (0.4)	2.8		
Educational level			40.159^a^	< 0.001
Primary school or below	67 (25.9)	2.96 ± 0.32		
Junior high-school	98 (36.8)	2.88 ± 0.36		
Senior high-school	59 (19.7)	2.56 ± 0.38		
College/university	58 (17.4)	2.30 ± 0.29		
Postgraduate or above	1 (0.3)	2.04		
Employment status			− 1.157^b^	0.248
Employed	79 (27.4)	2.66 ± 0.47		
Other	204 (72.6)	2.73 ± 0.41		
Average family income (CNY)			30.817^a^	< 0.001
< 1000	27 (10.7)	3.05 ± 0.30		
1000 ~ 2999	112 (41.4)	2.83 ± 0.33		
3000 ~ 5000	78 (27.5)	2.71 ± 0.47		
> 5000	66 (20.4)	2.36 ± 0.33		
Health problems			− 1.394^b^	0.164
No	222 (77.9)	2.69 ± 0.42		
Yes	61 (22.1)	2.78 ± 0.45		
Relationship with patients			4.579^a^	0.001
Spouse	126 (45.9)	2.79 ± 0.45		
Child/children	115 (39.2)	2.61 ± 0.38		
Parents	8 (3.0)	2.89 ± 0.38		
Brothers and sisters	18 (6.7)	2.85 ± 0.28		
Others	16 (5.3)	2.52 ± 0.49		
Living with the patient			3.377^b^	0.001
Yes	181 (65.4)	2.77 ± 0.43		
No	102 (34.6)	2.60 ± 0.38		
Number of assisted caregivers			3.835^a^	0.005
0	125 (45.7)	2.80 ± 0.37		
1	109 (37.1)	2.61 ± 0.43		
2	39 (13.9)	2.74 ± 0.49		
≥ 3	10 (3.3)	2.50 ± 0.50		

*UEBMI*, Urban Employees’ Basic Medical Insurance; *URRBMI*, Basic Medical Insurance for Urban and Rural Residents; *SMI* + *CMI*, Social Medical Insurance + Commercial Medical Insurance; a, *F*; b, *t*

### Supportive care needs level

The score of SCNS-P&C of the 283 CRC caregivers was 2.71 ± 0.42, which was at the medium level. The score of the 4 dimensions was presented in Table 2. The score of each item on the scale was sorted, and the 10 items with the highest score are listed in Table 3.

**Table 2:** SCNS-P&C total scores and each dimension scores (*n* = 283)

Variable	Number of items	Items average score ($\overline{x }$±s)
HCSIN	16	3.09 ± 0.64
PEN	8	2.49 ± 0.52
CRN	10	2.62 ± 0.40
SSWN	11	2.39 ± 0.48
Total score	45	2.71 ± 0.42

*HCSIN*, Health Care Service & Information Needs; *PEN*, Psychological & Emotional Needs; *CRN*, Communication & Relationship Needs; *SSWN*, Social Security & Work Needs

**Table 3:** Top 10 items with the highest score for SCNS-P&C (*n* = 283, points, $\overline{x }$±s)

Items	Items average score ($\overline{x }$±s)
31. Management of disease recurrence concerns	4.19 ± 0.77
2. Obtain information about the patient's prognosis or possible outcome	4.16 ± 0.78
41. If the patient does not recover as you expect	4.01 ± 0.94
6. Obtain information about the benefits and side effects of treatment	3.88 ± 0.97
14. Reduce stress in patients' lives	3.59 ± 0.87
5. Obtain relevant information about the patient's possible physical needs	3.53 ± 0.87
22. Help is needed to reduce the impact of your work or daily activities as a result of caring for patients	3.49 ± 1.17
10. An opportunity to discuss your concerns with your doctor	3.48 ± 0.89
13. Ensure that comments about patient care are addressed appropriately	3.48 ± 0.89
23. Find out about financial support and government benefits for you or your patient	3.44 ± 1.50

### Univariate analysis

Univariate analysis revealed that duration of illness, tumor stage, medical insurance, caregiver age, place of residence, educational level, average family income, relationship with patients, living with the patient or not, and number of assisted caregivers were influencing factors for the SCN of caregivers of colorectal cancer patients (*P* < 0.05) (Table 1).

### Correlation analysis

The total caregiver preparedness score of 283 CRC caregivers was 26.00 ± 4.81 points (range 13 ~ 39), which was in the middle level. The score of benefit finding was 57.35 ± 18.07 points (range 26 ~ 105), which was at the moderate level. The score of financial toxicity was 20.01 ± 9.74 points (range 3 ~ 41), which was high. The SCN was negatively correlated with caregiver preparedness (*r* =  − 0.555, *P* < 0.001), benefit finding (*r* =  − 0.534, *P* < 0.001), and financial toxicity (*r* =  − 0.615, *P* < 0.001). The correlation of each dimension score and the total score of SCNS-P&C of caregivers with caregiver preparedness, benefit finding, and financial toxicity are presented in Table 4.

**Table 4:** Correlations between SCNS-P&C, PCS, BFS, and COST-PROM (*n* = 283)

Variable	HCSIN	PEN	CRN	SSWN	Total score
Caregiver preparedness	− 0.497**	− 0.448**	− 0.461**	− 0.338**	− 0.555**
Benefit finding	− 0.358**	− 0.605**	− 0.508**	− 0.370**	− 0.534**
Financial Toxicity	− 0.515**	− 0.399**	− 0.457**	− 0.563**	− 0.615**

*HCSIN*, Health Care Service & Information Needs; *PEN*, Psychological & Emotional Needs; *CRN*, Communication & Relationship Needs; *SSWN*, Social Security & Work Needs; **, *P* < 0.001

### Multiple linear regression analysis

Multivariate regression analysis was performed with the SCN of caregivers as the dependent variable and the items with statistical differences in the univariate analysis, caregiver preparedness, benefit finding, and financial toxicity as the independent variables. Multivariate regression analysis suggested that duration of illness, tumor stage, the age and educational level of caregivers, caregiver preparedness, benefit finding, and financial toxicity were the influencing factors of caregiver SCN of caregivers (*R*^2^ = 0.574, *F* = 23.337, *P* < 0.001) (Table 5).

**Table 5:** Regression of SCN on critical explanatory factors (*n* = 283)

Variable	*β*'	Std. error	*Β*	*t*	*P*
Constant	175.378	7.081	—	24.768	< 0.001
Duration of illness	− 2.971	0.846	− 0.167	− 3.511	0.001
Tumor stage	6.697	1.535	0.187	4.364	< 0.001
Caregiver age	− 4.430	1.424	− 0.155	− 3.110	0.002
Educational level	− 4.970	1.213	− 0.279	− 4.097	< 0.001
Caregiver preparedness	− 0.777	0.227	− 0.195	− 3.422	0.001
Benefit finding	− 0.125	0.059	− 0.118	− 2.126	0.034
Financial toxicity	− 0.713	0.145	− 0.364	− 4.911	< 0.001

*β'*, Coefficient; *Std. Error*, standard error; *β*, standardized coefficient; *R*^2^ = 0.574; *F* = 23.337; *P* < 0.001

## Discussion

In this cross-sectional study, the Supportive Care Needs Survey-Partners and Caregivers (SCNS-P&C) was used to measure the SCN level of caregivers of CRC patients. Through investigating 283 CRC caregivers, we found that the SCN score of was 2.71 ± 0.42, slightly higher than the result (2.13 ± 0.61) recorded in a relevant study conducted by Liu in caregivers of different types of cancers [28]. In addition, among the scores of the four dimensions of SCNS-P&C, two factors (communication & relationship and psychological and emotional needs) were significantly higher than the results reported by Liu [28]. This is probably because CRC caregivers experienced a comparable embarrassment and stigma (caused by colostomy) with patients, which was notably higher than caregivers of other cancer patients [8]. In addition, it should be noticed that caregivers of CRC have to deal with changes in intestinal function, diet, and nutrition, especially colostomy care, which requires professional knowledge and skills and great patience [7]. All of these lead to a higher level of SCN for caregivers.

In this study, the dimension of health care services and information needs has the highest score, and caregivers were most concerned about “managing disease recurrence”, consistent with the results from a previous study [20]. The findings highlighted that CRC caregivers often feel uncertain about the future, have difficulty managing their fear of cancer progression and recurrence, and have a high psychological burden. Previous studies have shown that CRC caregivers in China generally have a heavy caring burden (e.g., including multiple burdens such as physical, psychological, economic, and social burdens) [7]. The heavier burden will harm the caregivers’ physical, mental, and social functions, increase the risk of disease, and further affect the patient’s psychological state and quality of life. Therefore, in clinical practice, medical staff should pay more attention to caregivers of CRC patients by regularly communicating with them and evaluating their needs through the SCNS-P&C scale to timely identify caregivers with high SCN and provide corresponding interventions (e.g., psychoeducational intervention [29] and problem-solving therapy [30]).

Our study demonstrated that middle-aged and young caregivers (40 ~ 60 years old) have more SCN than caregivers of other ages. This is consistent with a previous survey [31]. Middle-aged and young caregivers generally have more social roles (e.g., workplace duties, looking after families), requiring more social security support [31]. However, older caregivers usually have less workload and can receive help from their children. This reduces the care burden and results in a lower level of SCN. Hence, the SCN in this study appeared to be more in the middle-aged and young caregivers.

Additionally, this study showed that caregivers with a lower educational level have more SCN, which is inconsistent with the results of Niu et al. [32]. The differing results may be because caring for CRC patients requires a high level of knowledge and skills for caregivers. However, poorly educated caregivers generally have low health information literacy levels and a limited understanding of disease knowledge and information. As a result, they cannot effectively receive information from medical staff to participate in medical decision-making [41]. On the contrary, highly educated caregivers have a higher ability to acquire, understand, and apply information but also have certain advantages in communication skills and access to social resources, so their level of SCN is lower. This inconsistent evidence on the relationship between the educational level and SCN in cancer caregivers for further validation.

This study showed that the shorter treatment period and the higher tumor stage of CRC patients were significantly associated with the SCN of caregivers. In this study, nearly half of the caregivers cared for patients with a disease duration of 1 to 6 months, and their SCN score was 2.76 ± 0.40, higher than the average. Previous longitudinal studies suggested that, with the progress of treatment, there is a dynamic reciprocal relationship between caregivers’ mental health and caregiving burden. Reducing the caregiving burden by accumulating disease knowledge, nursing skills, and experience will gradually reduce their SCN [33]. In addition, our study found that the higher the tumor stage of CRC patients, the more SCN of the caregivers. As demonstrated by a recent study, CRC patients with higher tumor stages had poor physical and mental states and more adverse reactions. These factors led to more nursing problems for caregivers and caused increased care burden and the SCN [20]. A Taiwan study also showed that patients in poor physical conditions greatly depend on caregivers [34]. Therefore, we need to pay more attention to caregivers looking after patients with shorter treatment cycles and later tumor stages. These caregivers should be provided with additional physical and psychological support by actively and regularly assessing their needs.

Our study found that lower caregivers’ preparedness was associated with more SCN of caregivers, which is consistent with some previous studies [35], which suggested that caregivers of advanced cancer patients had a low level of preparedness, poor ability to cope with emergencies, a relative lack of nursing skills, and therefore, severe care burden and psychological distress [36]. Another study also showed that transitioning from hospital to home is critical [16]. During this transition period, caregivers assume a lot of responsibilities and burdens. Unprepared caregivers have low self-efficacy, poor role adaptation, and an inability to deal with various problems, increasing their SCN and reducing their well-being and overall quality of life [16]. This is a reminder that we should attach importance to the preparedness assessment of CRC caregivers, identify caregivers with low preparedness early, and provide targeted interventions to help them adapt to the role of caregivers as soon as possible, improve nursing skills, reduce psychological burden, and thus reduce the level of SCN.

The benefit finding was negatively associated with SCN, as shown in our study (*r* =  − 0.534, *P* < 0.001), which is consistent with the results of a previous study [37]. We also found lower benefit findings associated with more psychological and emotional needs of SCN (*r* =  − 0.605, *P* < 0.001). A study found that caregivers with a high benefit finding have a higher buffer capacity for negative emotions such as anxiety, depression, or uncertainty, a lower level of psychological distress, and a higher ability to adapt to their roles, thereby reducing their caring burden and improving their quality of life [18]. Another study showed that the benefit finding could encourage caregivers to cope with heavy care tasks positively, strengthen their psychological bearing capacity, enhance the intimate relationship with patients, enhance social support, and reduce SCN [38, 39]. This is a reminder that we should help CRC caregivers face crises and challenges positively, encourage them to accept problems and difficulties, bring in openness to external social relationships, and find meaning in the care process.

According to our results, lack of financial support was a key factor associated with SCN of the caregivers of CRC patients. We found that the financial toxicity score of caregivers of CRC patients was 20.01 ± 9.74, and 77.39% of caregivers reported high-level financial toxicity. Financial toxicity was negatively associated with the SCN of caregivers (*r* =  − 0.615, *P* < 0.001). It was also observed that caregivers with high-level financial toxicity had more social security and work needs. “Poverty reinstatement due to illness” and “poverty due to illness” were standard in China and were known by the patient and their caregivers [40]. Van Hof et al.’s study showed that continuous financial problems in the care process often led to more unmet SCN of caregivers [41]. Previous studies pointed out that employment disruption of caregivers exacerbated existing financial challenges, and caregivers’ division of labor between caregiving and providing financially led to heightened psychological distress [42]. These findings are in line with our study results. Therefore, it is necessary to strengthen the construction of a financial support and security system to enrich the external resources available to caregivers. Notably, medical staff should pay more attention to the treatment costs and family economic status of patients and caregivers, assess the financial toxicity of caregivers in real-time, identify caregivers who need help, and provide them with evidence such as medical cases and daily medical consumption list according to needs, and help them understand the support policies such as medical insurance reimbursement and serious illness medical assistance. It is crucial to encourage them to seek help from relatives, friends, and social welfare institutions in time.

## Limitations

There are several limitations regarding the generalizability of our study. Firstly, it was a single-centered study design. All the caregivers under investigation were recruited from the same hospital. Further studies with multi-center, larger sample sizes and universal coverage of areas are needed to validate our findings. However, caregivers with different ages, educational, and financial backgrounds were included in the study. Secondly, this cross-sectional design analyzed the SCN of CRC caregivers and related factors at only one point in the disease trajectory. Further studies examining prospective SCN of caregivers of CRC patients and their corresponding factor trajectory at different time points should be conducted to explore how SCN progresses across the caregiver’s trajectory.

## Conclusions

The caregivers of colorectal cancer patients in China have a moderate level of SCN. Most of the needs were in the “dimension of health care services & information” domain. Our results suggested that good caregiver preparedness, benefit finding, and financial toxicity could reduce these needs. In addition, the duration of illness, tumor stage, the age of caregivers, and education level were also principal factors that affect the SCN of caregivers of CRC patients. We suggest future studies should focus on large-scale prospective surveys to systematically assess the SCN of CRC caregivers and design effective interventions to solve the caregivers’ needs.

## Data Availability

The datasets generated during and analyzed during the current study are available from the corresponding author on reasonable request.
